# Cutting seton versus decompression and drainage seton in the treatment of high complex anal fistula: a randomized controlled trial

**DOI:** 10.1038/s41598-022-11712-9

**Published:** 2022-05-12

**Authors:** Qiuxiang Yu, Congcong Zhi, Lansi Jia, Hui Li

**Affiliations:** Department of Proctology, China-Japan Friendship Hospital, No 2 Yinghua East Street, Chaoyang District, Beijing, 100029 China

**Keywords:** Anal diseases, Medical research, Randomized controlled trials

## Abstract

This study aimed to compare the efficacy between decompression and drainage seton (DADS) and cutting seton (CS) in the treatment of high complex anal fistula. Patients were randomly assigned 1:1 to DADS or CS group. The primary outcome was the rate of wound healing. Second outcomes included time taken to return to work, postoperative pain, the severity of fecal incontinence and other complications. A total of 120 patients with a mean age of 39 years were included. There was no significant difference in the rate of complete wound healing at 1 year. The mean time taken to return to work was 5 ± 2 days in DADS group, shorter than CS group (10 ± 3, *p* < 0.001). Mean vaizey incontinence score and the post-operation pain in DADS group was significantly lower than CS group. No significant difference was found between two groups in the incidence of complications. DADS is as effective as Cutting seton for the treatment of high complex anal fistula but is associated with less postoperative pain and better sphincter function preserving.

## Introduction

Most cases of anal fistulas are superficial and can be treated with a simple fistulotomy. However, high complex anal fistula is associated with a large portion of external anal sphincter (EAS), thus remaining a challenge in surgery^1,2^. At present, seton techniques, including cutting seton (CS) and drainage seton, are still widely used in the treatment of high complex anal fistula. CS is used as a divider, cutting the muscle gradually as a result of pressure necrosis while healing the wound with granulation tissue. CS is based on the assumption that leads to a chronic inflammatory reaction and causes fibrosis consequently, preventing the sphincter from retraction when divided^3^. Despite of the increased cure rate, CS may be unsuccessful, resulting in impairment of the anorectal ring. Comparatively, drainage seton can better protect the sphincter complex, but it may fail as the incomplete opening of the deep intersphincteric space limits the effect of drainage of the wound. Decompression and drainage seton (DADS) is to make an incision in the internal sphincter with simplified marsupialization to remain the intersphincteric space open and decompressed. Then, drainage seton is introduced through the tract in the external sphincter to make the bidirectional full drainage of both sides of the external sphincter. This study aimed to evaluate the clinical efficacy and safety of DADS in treatment of high complex anal fistula.

## Results

### Patient characteristics

163 patients were initially enrolled, and 43 patients were excluded as they did not meet the eligibility criteria. 120 patients were included and randomly assigned 1:1 to DADS group or CS group. According to Garg classifications^4^, there were 53 males and 7 females in DADS group, aged 21–56 years (mean age 38.15 ± 11.67 years), including 43 cases of high linear transsphincteric fistula (grade III), 9 cases of high transsphincteric fistula with either abscess, multiple or horseshoe tract (grade IV) and 8 cases of suprasphincteric fistula (grade V). There were 51 males and 9 females in CS group, aged 21–60 years (mean age 39.95 ± 13.03 years), including 46 cases of high linear transsphincteric fistula (grade III), 7 cases of high transsphincteric fistula with either abscess, multiple or horseshoe tract (grade IV) and 7 cases of suprasphincteric fistula (grade V). There were no significant difference between two groups in age, sex and fistula type as shown in Table 1.

**Table 1:** Basic characteristics of patients in two groups.

Items	DADS	Cutting seton	t/x^2^	*p*
	n = 60	n = 60		
Age	38.15 ± 11.67	39.95 ± 13.03	− 0.797	0.786
Gender (F/M)	7/53	9/51	0.288	0.591
Garg classifications (Grade)				
High linear transsphincteric fistula (III)	43	46	0.418	0.811
High transsphincteric fistula with either abscess, multiple or horseshoe tract (IV)	9	7		
Suprasphincteric fistula (V)	8	7		

*DADS* decompression and drainage seton.

**Figure 1:**
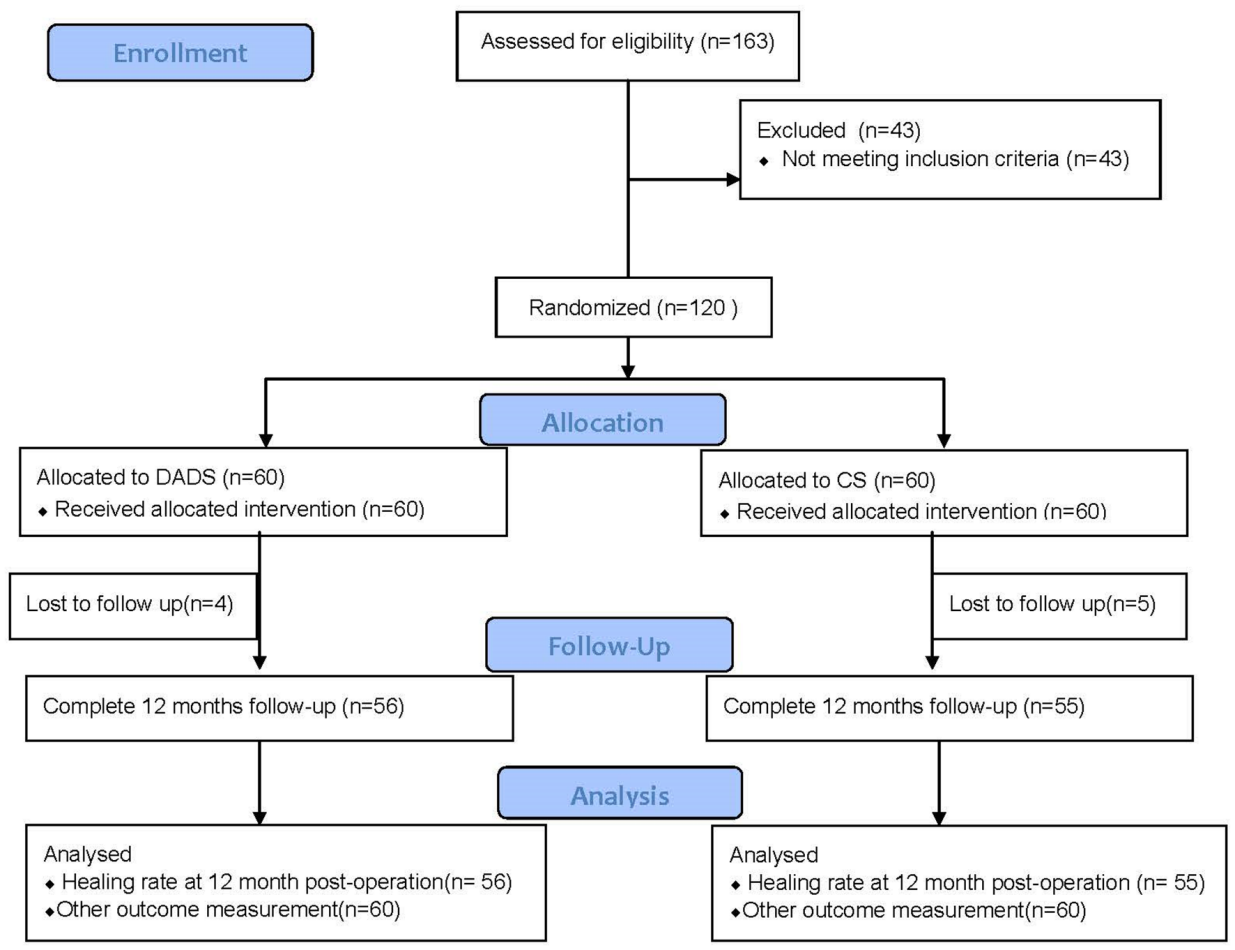
Study flow diagram. *Notes*: DADS = decompression and drainage seton, CS = cutting seton.

4 patients in DADS group and 5 in CS group were lost to follow-up in 12-month post-operation. All patients who were lost to follow-up were due to cell phone shutdown or cell phone number change. The study flow diagram was showed in Fig. 1.

### Primary outcome

#### Rate of healing

After 12-month follow-up, 5 patients didn't heal in CS group (9.1%, 3 for high horseshoe fistula, 2 for suprasphincteric fistula), and 4 in DADS group (7.1%, 1 for high horseshoe fistula, 3 for suprasphincteric fistula). There was no significant difference between the two groups in healing rate (*p* = 0.707) (Table 2).

**Table 2:** Comparison of efficacy measurements between two groups.

	DADS	Cutting seton	t/x^2^	*p*
**Primary efficacy measurement**				
Rate of healing at12 month post-operation	n = 56	n = 55		
	92.9%	90.9%	0.141	0.707
**Secondary efficacy measurement**				
Time of resumption to work (days)	n = 60	n = 60		
	5 ± 2	10 ± 3	− 10.742	0.000
Post-operation pain	n = 60	n = 60		
1 day VAS post-operation	4.0 ± 1.1	6.1 ± 1.3	− 9.552	0.000
1 week VAS post-operation	3.3 ± 0.8	4.2 ± 1.1	− 5.125	0.000
Vaizey anal incontinence score				
	n = 60	n = 60		
Pre-operation	0	0	–	–
Post-operation	0.17 ± 0.5*	2.15 ± 3.4**	4.416	0.000
Perfect continence (score = 0)	54 (90.0%)	35 (58.3%)	17.056	0.001
Mild incontinence (score 1–6)	6 (10.0%)	18 (30.0%)		
Moderate incontinence (score 7–12)	0	5 (8.3%)		
Severe incontinence (score 13–24)	0	2 (3.3%)		
Total complications	n = 60	n = 60		
	1 (1.7%)	5 (8.3%)	2.807	0.094

*VAS* visual analog scale, *DADS* decompression and drainage seton.

*p < 0.05 pre-operation VS post-operation, **p < 0.01 pre-operation VS post-operation.

### Secondary outcomes

#### Time taken to return to work

The mean time taken to return to work was 5 ± 2 days in DADS group, and 10 ± 3 in CS group. There was significantly difference between the two groups (*p* < 0.001).

#### Post-operation pain

The post-operation pain was measured by the visual analogue scale (VAS)^5^ after 1 day and 1 week post-operation. The mean VAS at 1 day post-operation in DADS group was 4.0 ± 1.1, which was significantly lower than CS group (6.1 ± 1.3, *p* < 0.001). The mean VAS in DADS group at 1 week post-operation was 3.3 ± 0.8, significantly lower than CS group (4.2 ± 1.1, *p* < 0.001).

#### Vaizey incontinence score

Before surgery, continence issues did not occur in all subjects (score 0). After operation, mean vaizey incontinence score^6^ was 0.17 ± 0.5 in DADS group, significantly lower than CS group (2.15 ± 3.4, *p* < 0.001). In DADS group, 54 cases (90%) had normal continence (score 0), 6 cases (10.0%) had mild incontinence (score 1–6). In the cutting seton group, 35 cases (58.3%) had normal continence (score 0), 18 cases (30.0%) had mild incontinence (score 1–6), 5 cases (8.3%) had moderate incontinence (score 7–12), 2 cases (3.3%) had severe incontinence (score 13–24). Significant difference was found between the two groups in the continence (*p* = 0.001).

#### Complications

5 patients (8.3%) in CS group had complications, and all 5 patients had urinary retention within 24 h post-operation. In contrast to DADS group, only 1 patient (1.7%) had urinary retention (*p* = 0.119). No postoperative hemorrhage occurred in both groups.

## Discussion

High complex anal fistula involves more external anal sphincter, the radical treatment of which may be associated with some disturbance of the continence state. Some innovative procedures aiming to preserve both anal sphincters completely including anal fistula plug^7^, rectal advancement flap^7^, video-assisted anal fistula treatment (VAAFT)^8^, and ligation of the intersphincter fistula (LIFT)^9^ may have a high risk of recurrence. Although these methods focus on closing the internal opening or ligating the tract in intersphincteric plane, the failure after these procedures were probably due to inadequate drainage of secondary extensions of the primary fistula track in the intersphincteric space^10^. Studies^11,12^ had found that most posterior high complex fistulas were associated with a primary or secondary lesion in the posterior deep space^12^, namely deep posterior intersphincteric space (DPIS)^12^.

In this study, DADS was similar to transanal opening of intersphincteric space (TROPIS), both of which attached great importance the role of intersphincteric lesions in the pathological development of complex anal fistulas. Nevertheless, the theoretical basis of the two procedure was different. Garg^13^ highlighted two important principles in TROPIS procedure: firstly, intersphincteric tract was like an abscess in a closed space, and secondly, ensuring continuous drainage was crucial for healing. As a result, in TROPIS, the portion of fistula inside the external sphincter (internal opening and the intersphincteric portion of the fistula tract, including secondary tracks) was laid open by incising internal sphincter over the fistula to achieve continuous drainage. Besides, the outside portion of fistula the external sphincter could be managed by excision of the tracts tunnel-Like, curettage and insertion of a tube, laser excision, etc. But why not perform those (excision of the tracts tunnel-Like, curettage and insertion of a tube, laser excision) to manage the portion of fistula inside the external sphincter, which could open the end near the anal verge of the tract so as to get continuous drainage theoretically and protect internal sphincter meanwhile?

**Figure 2:**
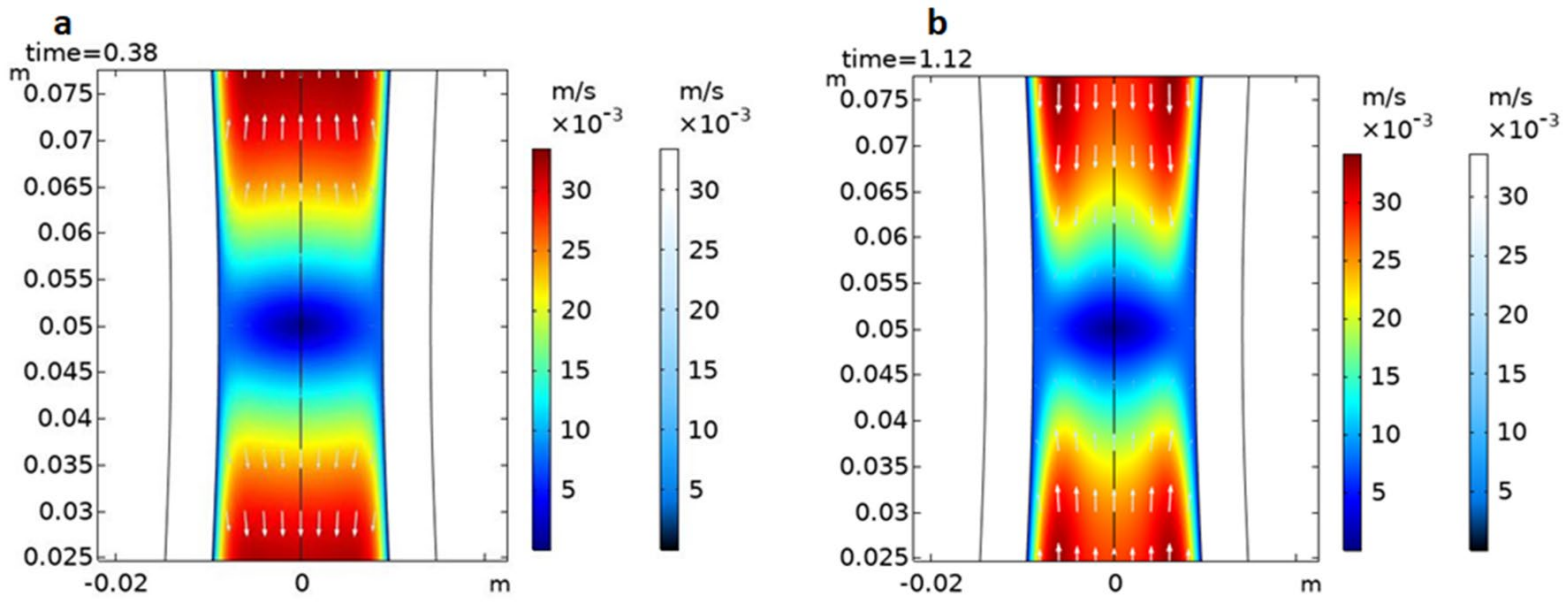
Two-dimensional flow field Cloud map. *Note*: Numerical simulation illustrated the calculated results of Liquid flow direction (white arrow),flow velocity, coupling surface pressure and tube wall shape variables at two time steps (0.38, 1.12 s), The former (**a**) represents fluid is squeezed out when the sphincter is contracted, the latter (**b**) represents fluid is inhaled when the sphincter relaxes.

We hypothesize that the sepsis in the deep intersphincteric space is affected by the activities of the internal and external sphincter. According to the fluid–structure interaction, when the sphincter contracts, the fluid in the intersphincteric space can be squeezed out. On the contrary, the fluid can be inhaled when the sphincter relaxes, which was illustrated using numerical simulation method by COMSOL Multiphysics® 5.6 (Fig. 2). The effect of sphincter contraction/relaxation may cause vortex and reflux, which may cause uncontinuous drainage. This can explain the high recurrence rate of other sphincter-preserving procedures including curettage fistula in the deep intersphincteric space due to the inability to ensure continuous drainage of the intersphincteric space.

DADS is to spare the fistula in intersphincteric space from the dynamic high pressure between internal and external sphincter to achieve continuously drainage by incising internal sphincter over the fistula tract in intersphincteric space, and the suture 2–3 stitches on both sides of the incision. The fistula “outside the external sphincter” is incised from the external opening to external sphincter, the seton is then inserted through the curetted tract in the external sphincter so that both sides of the external sphincter can achieve better drainage of pus in fistula-related spaces, decreasing the risk of persistence or recurrence.

There are several sphincter-sparing techniques that aim to tackle the tract in the intersphincteric space, such as fistula plug, VAAFT, and LIFT. Because the high complex fistulas usually have a secondary lesion and the tracts are deep in the intersphincteric space, it is hard to eradicate the pus completely, which causes the non-negligible recurrence rate. The Tunnel-Like Fistulectomy Plus Draining Seton Combined with Incision of Internal Opening of Anal Fistula (TFSIA), reported by Yan et al., is similar to the present technique^14^. Both attach great importance to the role of infection tissue in the intersphincteric space as the recurrence of complex anal fistula. The recurrence rate reported in the TFSIA is 12.5%.There is another external anal sphincter-sparing seton trail, also paying attention to the secondary extensions of the primary fistula track in the intersphincteric space^15^, which seton is rerouted and tightened around the IAS only and is allowed to cut through the sphincter fibers until it fell spontaneously. The persistence or recurrence rate of this procedure is 8.3%.

During the 12-month follow-up after surgery, 1 case relapsed within 6 months and 4 cases relapsed within 9–12 months in CS group. Comparatively, 4 cases in DADS group relapsed, thus no significant difference between two groups was found in the healing rate. The time taken to return to work was significantly shorter in DADS group than CS group. This was consistent with the significant difference in postoperative pain in the DADS group compared with the CS group. This might due to the fact that the internal sphincter was cut directly with electrocautery in DADS group, while the internal and external sphincter was cut by seton in the CS group. The thicker the tissue was cut, the more sever the pain was. Although CS group only tightened the seton once after the operation, the pain during the tightening process should not be underestimated. The scores of anal incontinence in DADS group were lower than those in CS group. These might due to the fact that the external anal sphincter in DADS group was fully protected.

There are some limitations in this study. Firstly, this was a single-center trial involving relatively few patients. Secondly, this trial was single-blind, so the bias caused by the researcher's subjective factors cannot be avoided. Thirdly, it is known that clinical healing (closure of external opening) may occur without fistula healing in intersphicteric space, resulting in recurrence later. However, postoperative MRI examinations to confirm healing was not asked in our study due to economic factor. Therefore, a multicenter, randomized, double blind clinical trial consisting of a large number of patients with longer follow-up is needed to determine the initial positive results of the trial.

## Conclusion

DADS is a promising technique for the treatment of high complex anal fistula, is as effective as Cutting seton but is associated with less time taken to return to work, as well as less pain and better sphincter function preserving. Studies with longer follow-up and larger sample size are needed to ascertain the efficacy and safety of DADS.

## Methods

### Study design

This was a single-blind, randomized controlled trial, registrated in clinicaltrials.gov (NCT05087407) at 21/10/2021, with ethical approval from the Institutional Review Board of China-Japan friendship hospital (No. 2016-81). All subjects provided written informed consent. The authors asserted that all procedures in this trial was in accordance with the ethical standards and with the Helsinki Declaration.

### Participants

**Figure 3:**
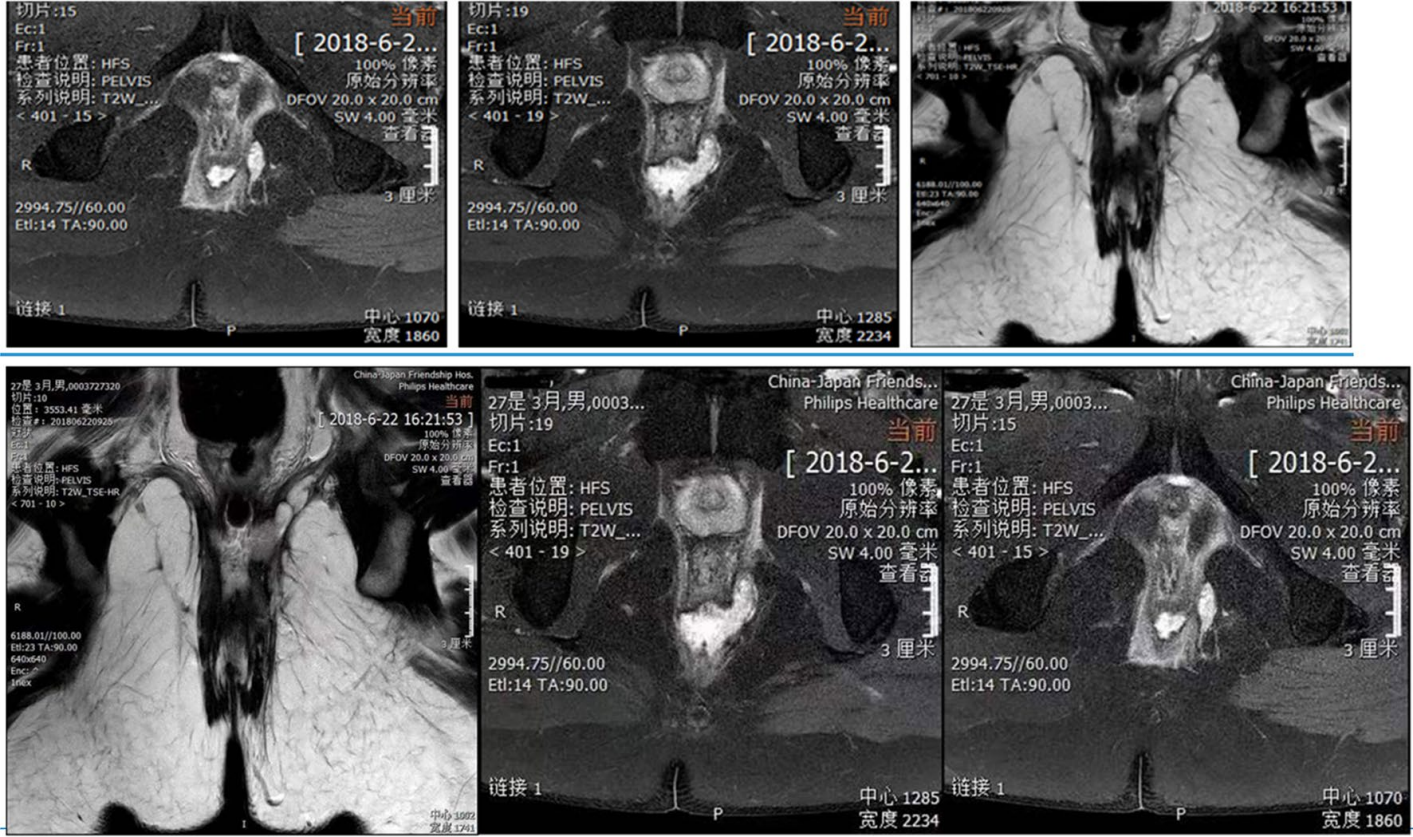
Suprasphincteric fistulas.

Participants were recruited in the department of Proctology, China-Japan friendship Hospital from December 2016 to December 2019. Patients aged between 21 and 60 years with high trans-sphincteric cryptoglandular anal fistulas (involving > 30% of the EAS muscles evaluated through magnetic resonance imaging (MRI) scan or endoanal ultrasonography)^10^, and/or suprasphincteric fistulas were enrolled, regardless of sex. The MRI pictures of suprasphincteric fistulas were shown in the Fig. 3. The following patients were excluded: (1) Low anal fistula; (2) Non-glandular anal fistulas, such as tuberculous anal fistula and Crohn's anal fistula; (3) Fecal incontinence; (4) A previous history of anal fistula surgical treatment; (5) Malignant tumors, mental illness or other reasons unable to cooperate with the treatment.

### Randomization and masking

Enrolled patients were assigned in a 1:1 ratio to DADS or CS group using a randomization software (www.randomization.com). The randomized code was generated in the randomization process and sealed in an envelope. In this single-blind clinical trial, the participants and statistician were both blind to the treatment, the operating surgeons were aware of the nature of the study and allocation of the groups. Once the blinding was broken, the patient was managed as fall-off.

### Preoperative assessment

Detailed history-taking, system examination and Vaizey incontinence score evaluation were asked prior to surgery^6^. Inspection of the perineum and anal verge was detected and the number and site of the external opening of the fistula were recorded to exclude the presence of associated anal conditions. Digital rectal examination was performed to exclude association of anorectal pathology. Patients were applied to endoanal ultrasonography or anorectal MRI to differentiate the type and complexity of the anal fistula, position of internal opening, and to detect secondary tracts and supralevator extensions.

### Procedures

Written informed consents were obtained from all patients after explaining possible benefits and complications of each technique. Subjects in both groups were given general anesthesia and placed in lateral position. A secondary fistula and the location of the internal opening of the anal fistula were determined by preoperative endoanal ultrasonography or anorectal MRI scan, as well as digital examination, probe exploration or injection of hydrogen peroxide or methylene blue during the operation. A malleable metallic probe was inserted into the external opening and gently guided until its tip came out of the internal opening. The subcutaneous part of the fistula tract (lying outside the EAS) was excised by electrocautery and the remaining part and any secondary extensions were thoroughly curetted. Then seton (0- silk suture) was inserted in the fistula tract.

**Figure 4:**
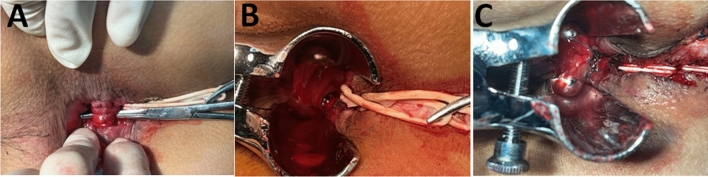
Decompression and drainage seton. *Notes*: (**A**) After the seton was rerouted by dissection in the intersphincteric plane, and was tied loosely around the EAS.A curved artery forceps was inserted through the internal opening to the intersphincteric part of the fistula tract. (**B)** The internal sphincter was cut with electrocautery to open the fistula tract. (**C)** Wound were sutured along the edge of fistula tract using interrupted absorbable polyglactin 3–0 sutures to marsupialize the operative wound from distal to proximal.

For DADS group, after inserted in the fistula tract, the seton was rerouted by dissection in the intersphincteric plane, and was tied loosely around the EAS. A curved artery forceps was inserted through the internal opening to the intersphincteric part of the fistula tract. The mucosa and the internal sphincter over the forceps were incised by electrocautery. If secondary fistula existed, curved forceps were used to reach the top of the fistula in the high inter-sphincteric and the supralevator spaces, and the internal sphincter was cut by electrocautery to open the fistula tract. Marsupialization was performed, as wound were sutured along the edge of fistula tract from distal to proximal using interrupted absorbable polyglactin 3–0 sutures, which would prove difficult proximally due to friable anorectal mucosa. Hemostasis was achieved by electrocoagulation (Fig. 4).

For CS group, after the seton was inserted in the fistula tract, the anal mucosa and perianal skin on the fistula surface was cut. The seton was tied tightly around the internal anal sphincter and EAS.

The drainage seton in DADS group was removed after the electrocauterized wound in rectum healed; no subsequent intervention were made in CS group until the seton to fall off on its own. Tighten the seton if it did not fall off 3 weeks after the operation and have a double check 2 weeks later. If the seton did not get removed spontaneously, the remaining sphincter would be cut off.

### Follow up time point and outcomes

All patients were followed up in outpatient at 1 week, 2 weeks, 1 month, 2 months, 6 months and 12 months postoperatively. During each follow-up visit the degree of incision healing, recurrence, complications, and continence state were recorded.

#### Primary outcome

Rate of complete healing: complete healing was defined as complete epithelialization of the wound, with no evidence of external fistula opening or perianal discharge under physical examination. The final curative effect was estimated 12 month post-operation.

Secondary outcomes include:

- Time taken to return to work (days).
- Postoperative wound pain was assessed by VAS from 0 to 10 at 1 day and 7 days after surgery, where 0 indicated no pain and 10 the worst possible pain.
- Postoperative complications within 7 days after surgery (bleeding and urinary retention).
- The continence state was assessed with Vaizey incontinence score before and 6 months after surgery (six parametersincluded: gas incontinence, liquid and solid incontinence, lifestyle changes, pad wearing, antidiarrheal drugs consumptions, and the ability to delay bowel movements by 15 min. No anal incontinence is 0 point, and complete incontinence is 24 points).

### Sample size calculation

The sample size was estimated using non-inferiority analysis by primary outcome (healing rate) in each group. Based on a previous study^16^ that regarded the CS as a treatment for anal fistula, we anticipated healing rate in the controlled group was 90% in the CS group, and 95% in the treatment group. The expected difference of healing rate in the two groups was 0.15 study power of 80% with an *α* set at 0.025, the sample size ratio between the treatment and the control group was 1:1. Assuming a conservative 20% proportion of losses, 58 patients in each group would be required.

### Statistical analysis

Stata 13.0 was used for data analyses. Mean ± SD was calculated to express continuous data, and the categorical variables were presented as number and percentage. *T* test was used to compare the continuous data (age, course of disease and time taken to return to work, etc.). *χ*^2^ test was used for categorical variables (rate of healing, gender, incidence of recurrence, etc.). All tests of effects were conducted using a two-sided *α* level of 0.05 and 95% confidence intervals.

### Ethics approval

This study was approved through the Institutional Review Board of China-Japan friendship hospital (No.2016-81).

### Consent to participate

An informed written consent was taken from each patient.

## Data Availability

The datasets analysed during the current study are available from the corresponding author on reasonable request.
